# Evaluation of corrosion performance of superhydrophobic PTFE and nanosilica coatings

**DOI:** 10.1038/s41598-022-20729-z

**Published:** 2022-10-12

**Authors:** Mohammad Haji-Savameri, Ahmad Irannejad, Saeid Norouzi-Apourvari, Mahin Schaffie, Abdolhossein Hemmati-Sarapardeh

**Affiliations:** 1 Department of Petroleum Engineering, Faculty of Engineering, Shahid Bahonar University of Kerman, Kerman, Iran; 2 Department of Petroleum Engineering, School of Chemical and Petroleum Engineering, Enhanced Oil Recovery (EOR) Research Center, Shiraz University, Shiraz, 7134851154 Iran; 3 Department of Materials Engineering and Metallurgy, Faculty of Engineering, Shahid Bahonar University of Kerman, Kerman, Iran; 4 Key Laboratory of Continental Shale Hydrocarbon Accumulation and Efficient Development, Ministry of Education, Northeast Petroleum University, Daqing, 163318 China

**Keywords:** Chemical engineering, Engineering, Materials science, Materials for energy and catalysis, Nanoscale materials, Structural materials

## Abstract

Corrosion protection of metals is of paramount importance in different sectors of industry. One of the emerging techniques to prevent or reduce the damaging effects of this phenomenon is to apply superhydrophobic coatings on the susceptible surfaces. In this study, corrosion protection of steel is investigated by fabricating superhydrophobic coatings, using one-step electrodeposition process of nanosilica hybrid film and spraying process of polytetrafluoroethylene (PTFE) on steel surface and also preparation of micro/nano-composite coatings. The anti-corrosion behavior of the nanosilica hybrid film and PTFE coating with two types of microparticles including Al_2_O_3_ powder and glass beads in primer layer, and overcoat layer with and without SiO_2_ nanoparticles is studied. TOEFL polarization and electrochemical impedance spectroscopy (EIS) tests are conducted on coated steel samples to examine their corrosion performance in 3.5 wt% NaCl solution at a temperature of 25 °C. The results showed that the combination of superhydrophobic properties and low conductivity significantly improves the corrosion resistance. Evaluating the effect of adding SiO_2_ nanoparticles to the overcoat layer in PTFE coating showed that the nanoparticles improve the corrosion resistance of PTFE coatings by sealing up some defects and pores in the coating. Investigation of corrosion resistance of coatings showed that, the corrosion resistance of nanosilica film is lower than that of PTFE coatings. The best sample obtained in this study, namely the PTFE coating with glass beads microparticles in primer layer and SiO_2_ nanoparticles in overcoat layer, reduced the corrosion rate by nearly 80 times.

## Introduction

Metal is one of the main materials in the human hands and its use in various industries is increasing day by day. They are used in various sectors of the industry such as construction (commercial building, housing, and roads), defense (firearms, ammunition, missiles, tanks, and jets), transportation (marine, aerospace, automobile), and medical (prosthetics, reconstructive surgery and biomedical implant)^1^. Metal structures and equipment are susceptible to corrosion when exposed to adverse environmental conditions and moisture. Corrosion causes loss of performance and ultimately destruction of equipment and metal structures. Surveys in the USA show that corrosion of steels and other metallic materials accounts for approximately 4–5% of the cost of gross domestic product (GDP)^2^.

Different methods have been used to prevent corrosion, the most important of which are: cathodic and anodic protection, corrosion inhibitors, and coatings^3–8^. Each of these methods has its advantages and disadvantages and may be used alone or in combination^9^. Coatings are generally substances used to create a barrier between the corrosive environment and the surface of the piece in question and protect metal parts from moisture, oxidation and chemicals^10^. For a long time, chromating and phosphating have been used as common methods to protect the surface of metals. But these two methods are not environmentally friendly. Toxicity and carcinogenicity of chromium (VI) have been proven for humans today, and phosphorous pollution is one of the important factors in contributing to water eutrophication^11,12^. The use of these materials to protect against corrosion of metals is prohibited in many countries. Much work has been directed towards developing other types of coatings. Different kinds of alternative materials, based on the use of the films of rare earth compounds^13,14^, sol–gel derived films^15–20^ and self-assembled layers^21,22^, have shown their ability to protect against corrosion. Studies have also shown that coatings with very low electric conductance such as non-conducting Al_2_O_3_, TiO_2_, SiO_2_ coatings, mix-oxides coating of Al_2_O_3_, TiO_2_, and SiO_2_ are very effective in protecting against corrosion^23,24^. The use of superhydrophobic coatings with contact angles (CA) higher than 150° and roll-off angles lower than 10° is an interesting approach to prevent metal corrosion, and have been followed in some research studies^25,26^. Drops slid on these surfaces as they form, and detach from the surface. Therefore, the contact time of the fluid drop (water or any corrosive fluid such as sulfuric acid) on the surface is dramatically reduced. Also, due to the roughness of the nanostructures on the surface, and the presence of air which is trapped between the cavities, the fluid contact with the corrosion-prone surface is reduced. Due to the simultaneous presence of these two effects (short contact time and low contact area), the corrosion resistance of metal surfaces covered with superhydrophobic coatings increases several times^25,27–29^. These coatings prevent the corrosion caused by electrolyte penetration into the metal substrate. Superhydrophobic coatings could be fabricated on many surfaces, especially the surfaces of metals and their alloys, such as Copper^30–32^, Aluminum^33–35^, Zinc^36,37^ and Magnesium^38,39^.

Although there are different fabrication methods for PTFE coatings such as spray, electrospray, chemical vapor deposition (CVD), etc., these methods often do not create the superhydrophobic surface or there are various operational limitations in construction of these coatings. For example, the electrospray method may lead to some macro-molecule degradation due to the variation in the operating parameters^40,41^. In some chemical vapor deposition reactions, it is very difficult to control the reactions and consequently the uniformity, and there is a possibility of unwanted reactions in this method, which may sometimes cause serious problems in the deposition process or inside the reactor. It is also possible for the substrate to be destroyed while using this method^42–44^.

In the current study, we produce PTFE superhydrophobic coatings on steel substrates for the purpose of corrosion protection. The PTFE superhydrophobic coating with hierarchical structure is produced by spraying on metal surface. The method which is used in our study to create PTFE coating is very simple and applicable to any type of surface and has no operational limitations associated with other methods. This coating also has superhydrophobic properties. In the construction of this superhydrophobic coating, in order to achieve a hierarchical structure, Al_2_O_3_ microparticles, glass beads microparticles and silica nanoparticles are used as materials with very low electric conductance. In order to evaluate the corrosion properties of PTFE coating, several superhydrophobic coatings with different specifications are produced and the effect of changing the type of microparticles in the primer layer and also the effect of the presence of nanoparticles in the overcoat layer on corrosion properties are investigated. Among various coating production methods to protect metal corrosion, the electrodeposition technique has been considered as a useful method for coating the metal surface, due to its advantages such as low-cost and the ability to apply on large scale surfaces as well as complex surfaces^45^. This technique has been the subject of much research and laboratory work. Since the comparison of candidate materials is one of the most basic steps in selecting the optimum material for engineering applications, we synthesize nanosilica superhydrophobic coatings via electrodeposition of organic/inorganic hybrid sol gel films from dodecyltrimethoxysilane (DTMS) and tetraethoxysilane (TEOS) mixed sol gel precursors, and we present a comparative study between the corrosion resistance of coatings made by spray and the electrodeposition methods. These coatings are made from low-cost, chemicals and especially from materials with very low electric conductance. In addition, in PTFE coating, the effect of parameters such as the type of microparticles used in the construction of the hierarchical surface and the effect of the presence of nanoparticles on corrosion properties is studied in details.

## Materials and experimental procedures

### Coating process

First, working electrodes with demension of 2.5 × 10 ×  0.1 cm^3^ were cut from a carbon steel sheet. The samples were then prepared with 80 grid emery papers, degreased with ethanol and finally washed with distilled water. After initial preparation, superhydrophobic PTFE composite coatings with two different types of microparticles, including Al_2_O_3_ and glass beads and silica nanoparticles, were generated on the carbon steel substrate. Coatings were applied on the samples by a pressurized pistol. While spraying the PTFE solution, the pressure was adjusted between 50 and 100 psi and the pistol head distance to the surface of the samples was about 20–30 cm. The coating was applied in accordance with the IPS standard. A radiant oven was used and an appropriate time and temperature for PTFE baking was obtained. According to the results of the experiments, suitable conditions for PTFE baking to create hydrophobic property were measured to be 410 °C for a duration of 30 min. The PTFE coating consists of two primer and overcoat layers. The overcoat material with commercial code W6622H-5161P and primer material with commercial code W6622H-5161T were purchased from Qingdao Kaimosi Chemical Co., Ltd. The microparticles used in the generation of PTFE composite coatings include Al_2_O_3_ microparticles (Asia Sanat Gangineh Trading Co., Tehran, Iran) and glass beads (Danehaye shishehie Co., Tehran, Iran) with the size of 77–82 microns. Silica nanoparticles with size of 40–50 nm were purchased from US Research Nanomaterials, Inc.

In this study, another superhydrophobic coating was also synthesized by direct electrodeposition of organic/inorganic hybrid sol gel films from DTMS and TEOS mixed sol gel precursors as a result of the co-generation of low surface energy and high roughness. The specifications of the produced coatings with their average thickness are presented in Table 1. In order to produce silica film coating, TEOS with purity of 98.5% (Sinopharm Chemical Reagent Co., Ltd., Shanghai, China) and a DTMS with a purity of more than 93% (Tokyo Chemical Industry Co., Ltd.) were used. Test solution precursors for electrodeposition operations include 2 ml of TEOS, 2 ml of DTMS, 80 ml of ethanol and 20 ml of 0.2 M KNO_3_. The pH of the sedimentation bath was maintained at pH = 4 and continously monitored by a digital pH meter (model W3B, BEL). Distilled water was also used to make the sol–gel solution. During the deposition process, the sedimentation bath was stirred by a magnetic stirrer in order to maintain the dispersion and uniformity of the material concentration in the sol–gel solution. The electrodeposition process was performed at ambient temperature and pressure. Graphite was used as an anode for the electrodeposition of nanosilica film coatings. The cathode and anode were placed 2 cm apart from each other in a 80 ml volume container before initiating the process of electrodeposition. The optimum current density and deposition time for nanosilica coating were determined as 0.3 mA/cm^2^ and 15 min, respectively. It should be noted that the synthesis of this coating was done at ambient pressure and temperature of 40 °C.

**Table 1:** Specifications of the fabricated coatings with their average thickness.

Sample number	Type of coating	Acronyms	Thickness (microns)
1	Nano Silica film coating	Nano Silica	40.15
2	PTFE coating with micro Al_2_O_3_ in primer layer and nano SiO_2_ in overcoat layer	Micro Al_2_O_3_—with nano SiO_2_	105.36
3	PTFE coating with micro glass beads in primer layer and without nano SiO_2_ in overcoat layer	Micro glass beads—without nano SiO_2_	115.91
4	PTFE coating with micro glass beads in primer layer and with nano SiO_2_ in overcoat layer	Micro glass beads—with nano SiO_2_	110.77

It is worth mentioning that the sol–gel process, which is also known as chemical solution deposition, is a wet chemical method that is widely used in engineering and materials science for the synthesis of various nanostructures. Therefore, the bonding between the nanosilica superhydrophobic coating components in this process, as is clear from the name of this process, is a chemical bonding. In the case of PTFE superhydrophobic coating, the first layer is a primer or a basecoat, followed by an overcoat layer. As a result of baking this type of coating, a strong bond is created between the metal surface, the primer layer, and the overcoat layer. Therefore, the connection between the various components of this type of coating is a chemical bonding, same as the nanosilica superhydrophobic coating.

Another important point to highlight is that both nanosilica film coating and PTFE coating with micro glass beads in the primer layer and with nano SiO_2_ in the overcoat layer (micro glass beads—with nano SiO_2_) have been used previously for experimental study and modeling of asphaltene deposition on metal surfaces^46^. In that research^46^, the synthesis method of nanosilica film coating and also PTFE coating (micro glass beads—with nano SiO_2_) were briefly described. The main discussion of that research^46^ was the application of the mentioned two types of coatings in reducing asphaltene deposits as one of the heaviest, polar, and most problematic deposits in crude oil. In the present study, in addition to the two coatings used in the previous study, other types of PTFE coating (including micro Al_2_O_3_—with nano SiO_2_ and micro glass beads—without nano SiO_2_) were synthesized and other characteristics of these coatings, such as the thickness of the coatings, water contact angle (WCA) and the sliding angle (SA) of the coatings, AFM parameters, energy dispersive X-ray spectroscopy (EDS) for nanosilica film coating, and the SEM images of the surface morphology of four types of coatings is presented in three different magnifications and in more detail. Also, in this study, for the first time, the ability of four types of coatings to reduce the corrosion rate has been discussed and compared with the without coating sample.

### Characterization of samples

**Figure 1:**
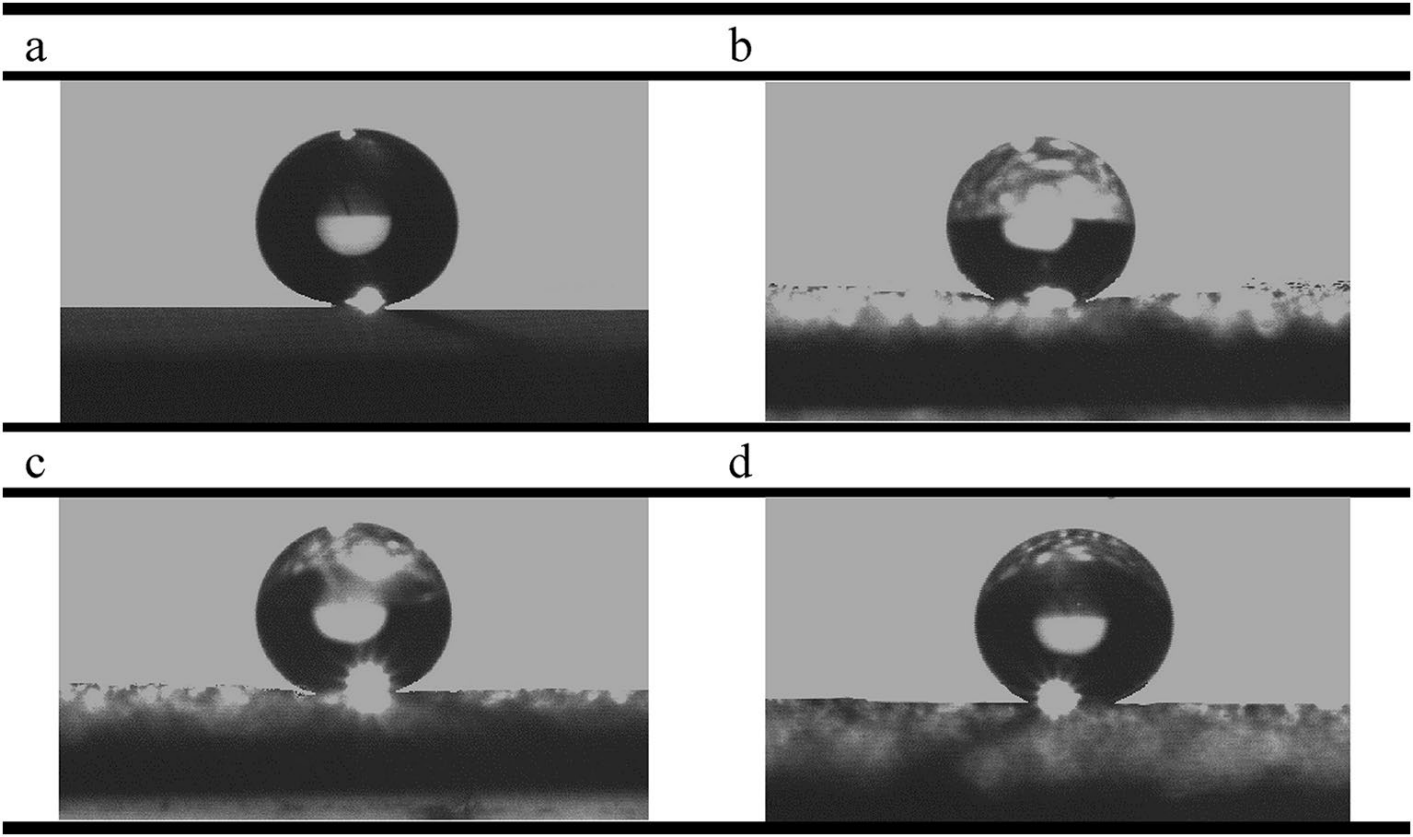
A water droplet on sample with PTFE and nanosilica superhydrophobic coatings: (**a**) nano silica^46^, (**b**) micro Al_2_O_3_—with nano SiO_2_, (**c**) micro glass beads—without nano SiO_2_, (**d**) micro glass beads—with nano SiO_2_^46^.

**Figure 2:**
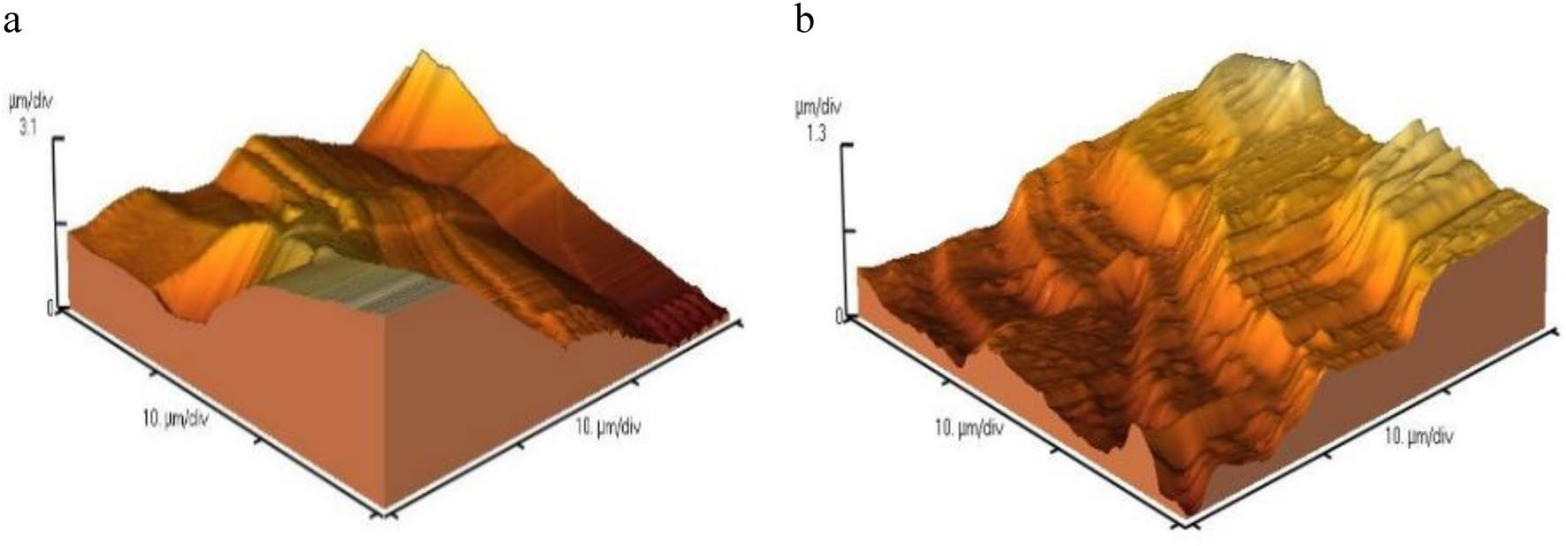
A 3D AFM images for (**a**) micro glass beads—with nano SiO_2_^46^, (**b**) nano silica^46^.

The surface morphology and chemical composition of coatings was investigated using field emission scanning electron microscope (FESEM, Hitachi S-4160, Japan) and Energy Dispersive X-Ray Spectroscopy (EDS, Ametek Element). The water contact angle and the sliding angle of coated and uncoated substrates were measured by CA measurement device (Drop Shape Analyzer-DSA100 KRÜSS GmbH, Germany). The contact angle reported in this research is the static contact angle. In this research, a 5 µl droplet^47–49^ was placed on the sample inside the device. Then, with a high-precision camera, the CA of the drop and its three-phase line was imaged at the point of contact with the surface. Finally, ImageJ software was used to calculate the angles. In a typical SA measurement, the coated or uncoated substrates were placed on a tilt stage at ambient pressure and temperature. A drop of water was then placed on the coated or uncoated substrates and allowed to equilibrate for ten seconds. Then the angle of the desired substrate was increased from the horizontal state (zero angle) at an approximate rate of half a degree per second. The angle at which the drop started to move was recorded as the sliding angle. The CA and SA reported in this study are the average of five measurements at different locations on the surface. Examples of CA images for PTFE and nanosilica superhydrophobic coatings are shown in Fig. 1. The roughness of the best sample of PTFE coating as well as nanosilica coating was measured using atomic force microscope (AFM) (CP II, Veeco—USA). The scanning range in AFM analysis was 10 × 10 µm^2^. Table 2 shows some roughness characteristics such as height roughness (Mean Ht), root mean square roughness (RMS Rough) and average surface roughness (Ave Rough) for the best sample of PTFE coating and also nanosilica coating. Figure 2 shows 3D roughness images for these two coatings.

**Table 2:** Calculated AFM parameters for nanosilica and PTFE coating with glass beads microparticles in the primer layer and with SiO_2_ nanoparticles in the overcoat layer.

Sample	RMS rough	Ave rough	Mean Ht
Nano silica	611.2 nm	539.8 nm	1.014 µm
Micro glass beads—with nano SiO_2_	1.255 µm	972.1 nm	2.926 µm

### Evaluation of corrosion performance

In TOEFL and electrochemical impedance spectroscopy (EIS) tests, EG&G M 263 (PARK) electrochemical measurement system was used to study the corrosion behavior of the coated surface and its analysis was performed using power suite software. The three-electrode system uses the coated sample as the working electrode, the calomel electrode as the reference electrode, and the Pt electrode as the counter electrode. The 3.5% NaCl solution is the electrolyte of this system. The rate of potential sweep in TOEFL test was 1 mV/s and the scanning potential range was 250 mV around OCP (open circuit potential). In EIS test the rate of potential sweep was equal to 1 mV/s and the scanning potential range varied from − 400 to 400 mV with respect to the OCP. Finally, the polarization and EIS tests were performed on coated samples with dimensions of 10 × 10 mm and the corrosion potential, corrosion current, and anodic and cathodic TOEFL constants were calculated and analyzed using CorrIII software. In this study, equivalent circuit simulation program, namely “ZSimpWin version 3.22”, was used for fitting of the experimental data, determination of the equivalent circuit, and EIS data analysis.

## Results and discussion

In order to investigate the corrosion properties of PTFE superhydrophobic coating, three types of coatings with different properties were made. Initially, superhydrophobic PTFE coating was made with Al_2_O_3_ microparticles in the primer layer along with SiO_2_ nanoparticles in the overcoat layer, and the effect of this superhydrophobic coating (with a hierarchical structure) on improving corrosion resistance was investigated. Two more samples of PTFE coating were then made with non-conducting glass beads microparticles in the primer layer, one of which has SiO_2_ nanoparticles in the overcoat layer and the other lacks of it. The morphology of coatings was first analyzed before presenting the results of the TOEFL and EIS tests.

### Morphology and chemical composition of coatings

**Figure 3:**
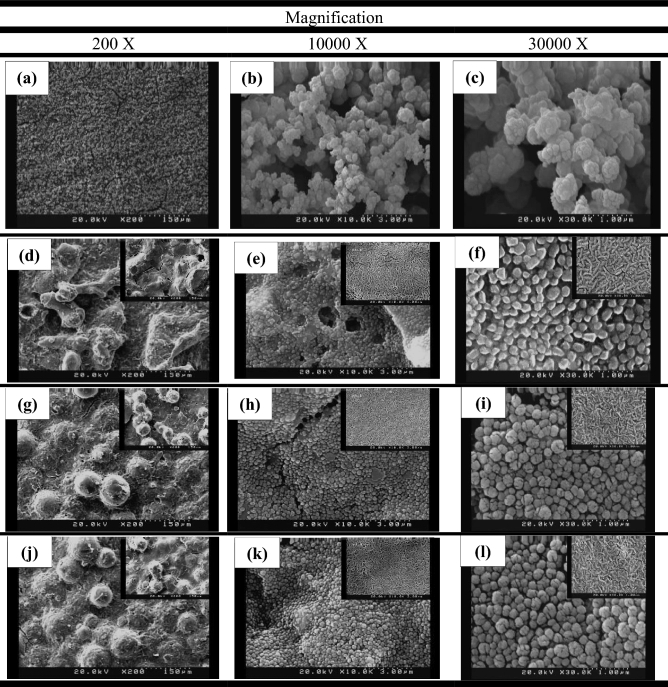
SEM images of nanosilica and PTFE coatings on MS substrate. (**a**–**c**) Nano silica coating^46^. (**d**–**l**) PTFE coatings includes morphological images of primer layer (large image), and primer and overcoat layers (small image): (**d**–**f**) Micro Al_2_O_3_—with nano SiO_2_, (**g**–**i**) Micro glass beads- without nano SiO_2_, (**j**–**l**) micro glass beads—with nano SiO_2_^46^.

**Figure 4:**
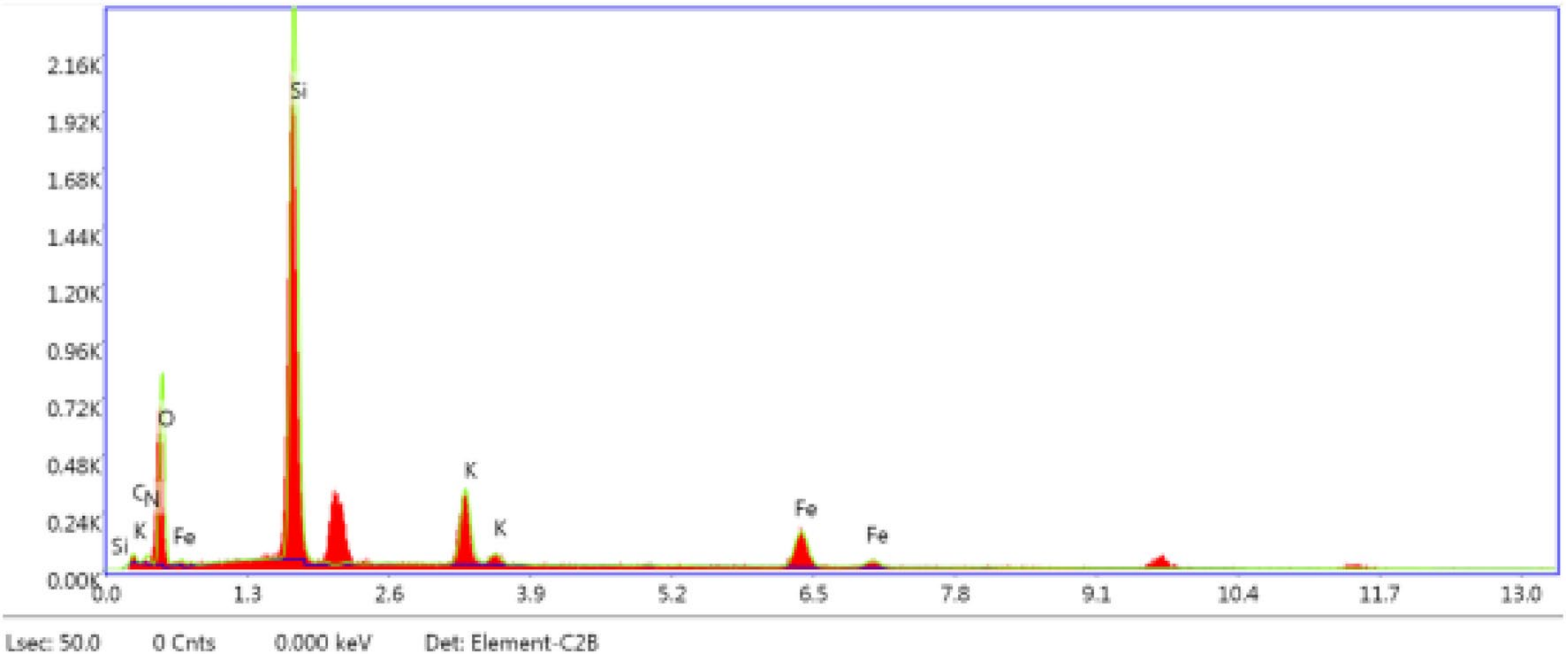
Energy dispersive X-ray spectroscopy of superhydrophobic nanosilica coating.

Figure 3 shows field emission scanning electron microscopy images of the coatings made in this study in three different magnifications. Figure 3a–c show the morphology of the nanosilica coating. It is observed that the nanosilica coating has a hierarchical structure. The air is trapped between the holes and heights of the structure and by limiting the contact of the passing fluid with the surface, the corrosion is decreased. The morphology of the coatings produced by the electrodeposition method strongly depends on the current density, electrolyte composition, temperature, deposition time, and the pH of the solution. Among these parameters, current density acts as a key factor in determining the structure of thin deposited layers^50,51^ in such a way that by adjusting the coating time and the current density, the size of the protrusions produced on the surface can be controlled. As the current density increases, the effect of cathodic polarization intensifies. As a result, the germination rate increases relative to the growth rate, which leads to the shrinkage of the structure^52^. In this study, the amount of current density and deposition time was obtained by trial and error. The superhydrophobic coating produced at a current density of 0.3 mA/cm^2^ and a duration of 15 min, had excellent stability over other produced samples and was therefore selected as a suitable coating for the corrosion process. Figure 3a shows that the surface of the coating is completely covered by spherical protrusions. The higher magnification images (Fig. 3b, c) show that on the spherical protrusions, many nanostructured protrusions are irregularly distributed. These results indicate that the nanosilica coating has a micro-nanometer hierarchical structure. This coating has a superhydrophobic property with a WCA of 166.24° and a SA of 0°. Figure 1a shows the CA of the water on the surface of this coating. Examination of Fig. 3d–l for PTFE coating also shows the hierarchical structure for the three coatings made in this study. In these figures, the larger images show the morphology of the primer layer with microparticles and the smaller images show the morphology of the overcoat layer on the surface of the primer layer. Figure 3d–f show PTFE coating with Al_2_O_3_ microparticles in the primer layer and SiO_2_ nanoparticles in the overcoat layer. This figure shows structures with angular shapes on a micrometer scale that have other protrusions on them. A comparison of the morphology of the overcoat layer and the primer layer in Fig. 3e and f shows that after applying the overcoat layer on the primer layer, the surface morphology gets a wormlike structure on a nanometer scale. Figure 3g–i show the surface morphology of PTFE coating with glass beads microparticles in the primer layer, which is coated by the overcoat layer without SiO_2_ nanoparticles. Figure 3g shows that this coating has a spherical structure on a micrometer scale. As can be seen in this figure, the placement of the overcoat layer on the primer layer results a wormlike structure at on the surface. Figure 3i–l shows the surface morphology of PTFE coating with glass beads microparticles in the primer layer and SiO_2_ nanoparticles in the overcoat layer. A comparison of morphological figures of this coating with PTFE coating containing glass beads microparticles in the primer layer and without SiO_2_ nanoparticles in the overcoat layer shows not much visual difference between these two coatings. A closer look at the overcoat layer in Fig. 3f, i, and l shows that the addition of SiO_2_ nanoparticles to the overcoat layer has no significant effect on the surface morphology of the overcoat layer. Investigating the corrosion behavior of these three samples can reveal the effect of adding SiO_2_ nanoparticles as well as the low conductivity of the materials used in the construction of rough surfaces in changing the corrosion process. These PTFE coatings have superhydrophobic properties with CAs of more than 150° and SAs of less than 5°. The exact details of the CA and SA of these PTFE coatings, along with the nanosilica coating and the uncoated sample, are listed in Table 3. Figure 1b–d show the CA for these PTFE coatings. According to the above explanations, all the coatings fabricated here have a hierarchical and rough structure. Roughness plays an important role in the wettability properties of the surface and thus improving corrosion resistance. As shown in Table 2, the surface roughness in the selected PTFE and nanosilica coatings is 1.255 µm and 611.2 nm, respectively. Figure 4 shows the energy dispersive X-ray spectroscopy of the nanosilica coating. As can be seen in this figure, the elements N, O, Si, K and Fe are present in this coating. The atomic percentages of N, O, Si, K and Fe are 4.3, 50.9, 32.9, 6.3 and 5.6, respectively. According to these values, the atomic oxygen/silica ratio is 1.54, which is close to 2. This emphasizes that the coating is made of SiO_2_.

**Table 3:** Water contact angle (WCA) and sliding angle (SA) of bare sample and sample with superhydrophobic coatings.

Sample	WCA (°)	SA (°)
Without coating	78.50	> 80
Nano silica	166.24	0
Micro Al_2_O_3_—with nano SiO_2_	152.45	5
Micro glass beads—without nano SiO_2_	154.40	3
Micro glass beads—with nano SiO_2_	155.32	3

### TOEFL extrapolation test

**Figure 5:**
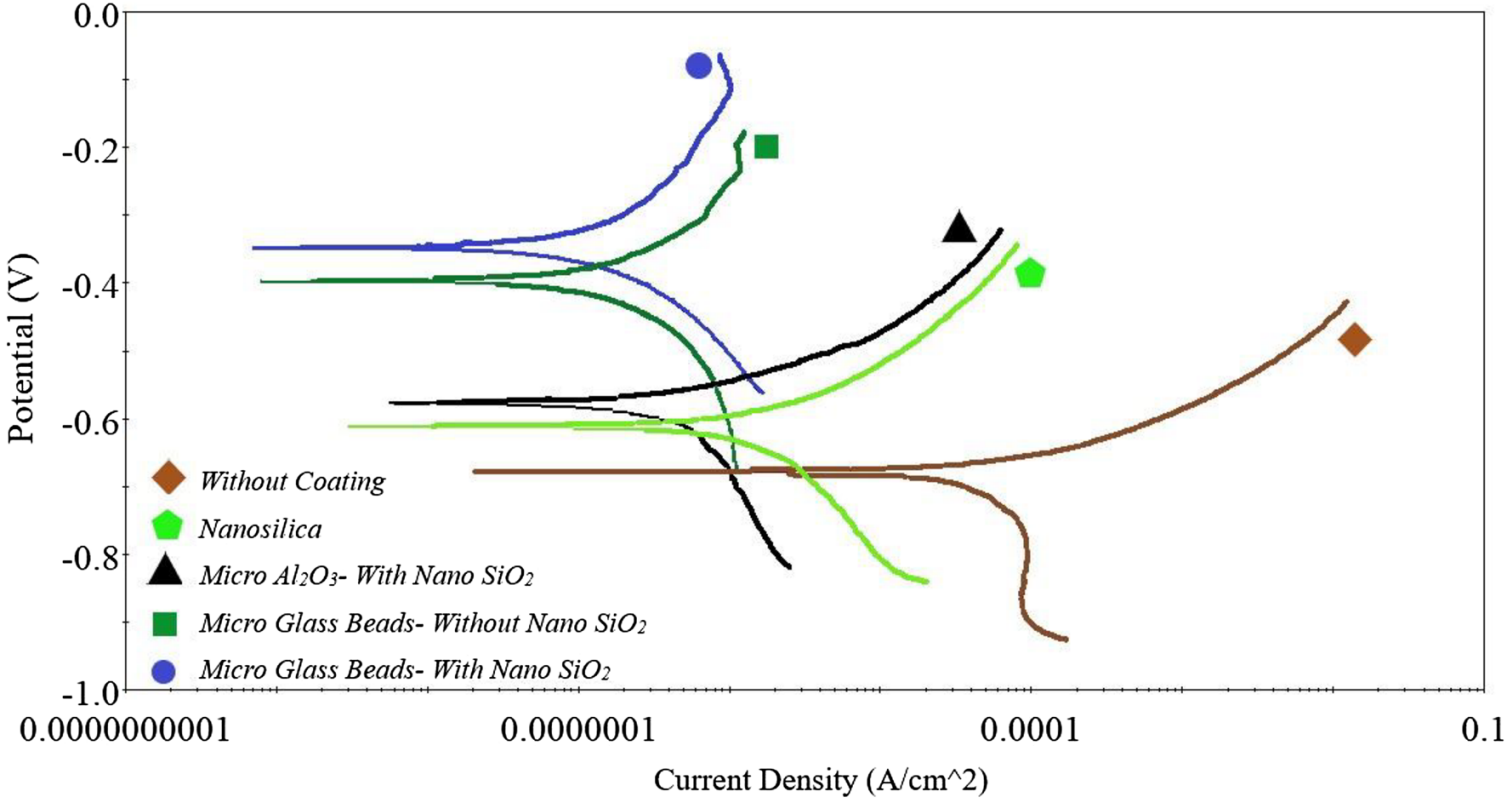
Comparison of potentiodynamic polarization curves of coated and uncoated samples.

Potentiodynamic polarization curves, for coated and uncoated substrates are shown in Fig. 5. From these curves, the corrosion potential, corrosion current density, and anodic and cathodic TOEFL constants can be extracted as listed in Table 4. The polarization resistance can be determined by the Stern-Geary equation (Eq. 1), which is based on the almost linear polarization behavior around the OCP point^53^.

1 $$ R_{p} = \frac{{\beta_{a} \beta_{c} }}{{2.303\left( {\beta_{a} + \beta_{c} } \right)}} \times \frac{1}{{I_{corr} }} $$

where, I_corr_ is the corrosion current density, R_p_ is the polarization resistance, β_a_ and β_c_ are the anode and cathode pabet. The TOEFL constant, which is a kinetic parameter, shows the rate of change of the anode and cathode potential. The higher the TOEFL coefficient, results in faster polarization and lower corrosion rate. Conversely, the lower TOEFL coefficient, results in slower polarization and greater corrosion^54^. By knowing the values of current density, the corrosion behavior of the samples can be evaluated. The lower the corrosion current density, the higher the polarization resistance and higher corrosion resistance of the coating^55^. As shown in Table 4, the corrosion resistance of all coated samples is much higher than that of uncoated samples and PTFE coatings with glass beads and Al_2_O_3_ microparticles perform better than silica film coating. This could be attributed to the lower thickness of the silica film coating compared to that of the PTFE coatings. Thicker coatings show lower corrosion current densities and consequently higher corrosion resistance^56^. The average thickness of the silica film coating is half of the average thickness of the PTFE coatings (Table 1). Coatings can increase corrosion resistance by increasing the charge transfer resistance in the metal-electrolyte interface, limiting the absorption of aggressive ions and increasing the substrate potential^57^. It can be seen in Table 4, that the PTFE coating with Al_2_O_3_ microparticles in the primer layer and SiO_2_ nanoparticles in the overcoat layer has a higher corrosion current density than the PTFE coating with glass beads microparticles (with and without SiO_2_ nanoparticles in the overcoat layer). This can be attributed to the semiconductor nature of the Al_2_O_3_ microparticles. Non-insulation Al_2_O_3_ microparticles has a higher corrosion density than insulating glass beads microparticles, and therefore its corrosion resistance is lower than that of PTFE coatings with glass beads microparticles.

**Table 4:** Potentiodynamic corrosion test results in 3.5% NaCl solution.

Sample	β_a_ (mV) anodic constant	β_c_ (mV) cathodic constant	i_corr_ (μA/cm^2^) corrosion current density	E_corr_ (mV) corrosion potential	R_p_ (Ωcm^2^) polarization resistance
Without coating	22.102	81.049	19.80	− 677.490	447.061
Silica film	77.991	151.413	1.294	− 611.432	16,544.886
Micro Al_2_O_3_—with nano SiO_2_	78.541	376.573	0.5750	− 577.656	55,598.428
Micro glass beads—without nano SiO_2_	247.750	314.784	0.3629	− 396.574	160,523.464
Micro glass beads—with nano SiO_2_	306.226	231.804	0.2560	− 346.737	220,922.779

A closer look at the results in Table 4 shows that, the PTFE coating with non-conducting glass beads microparticles in the primer layer and without nanoparticles in the overcoat layer, has much higher corrosion resistance than PTFE coating with semiconducting Al_2_O_3_ microstructures in primer layer and nanoparticles in the overcoat layer. The results of this study show that the low conductivity of the coating has a significant effect on reducing corrosion resistance. A comparison between the corrosion behavior of PTFE coating with glass beads microparticles in the primer layer and without SiO_2_ nanoparticles in the overcoat layer, and PTFE coating with glass beads microparticles in the primer layer and SiO_2_ nanoparticles in the overcoat layer, shows that adding nanoparticles in the overcoat layer, although does not make a difference in the surface morphology of the overcoat layer, but it has a great effect on improving corrosion resistance. The corrosion current density of PTFE superhydrophobic coating with glass beads microparticles in the primer layer and SiO_2_ nanoparticles in overcoat layer (Table 4) is about 0.2560 μA/cm^2^, which is approximately 1.41 times less than PTFE coating with glass beads microparticles in the primer layer and overcoat layer without SiO_2_ nanoparticles. Comparison of this coating with the uncoated sample shows a decrease in corrosion rate by more than 77 times. According to Table 4, it is observed that the corrosion potential has been transferred to noble values when the surface of the coating becomes superhydrophobic. Improving corrosion resistance can be attributed to the existence of holes and heights in the superhydrophobic surface, which causes air trapping between the depressions and limiting the surface exposure to corrosive solution. This superhydrophobic layer prevents the penetration of water and chloride-invading ions (Cl^−^) on the substrate surface and can ultimately play a much more effective protective role for the substrate. In the following sections, the effect of adding SiO_2_ nanoparticles on improving corrosion resistance for PTFE superhydrophobic coatings will be discussed further.

### Electrochemical impedance spectroscopy test

**Figure 6:**
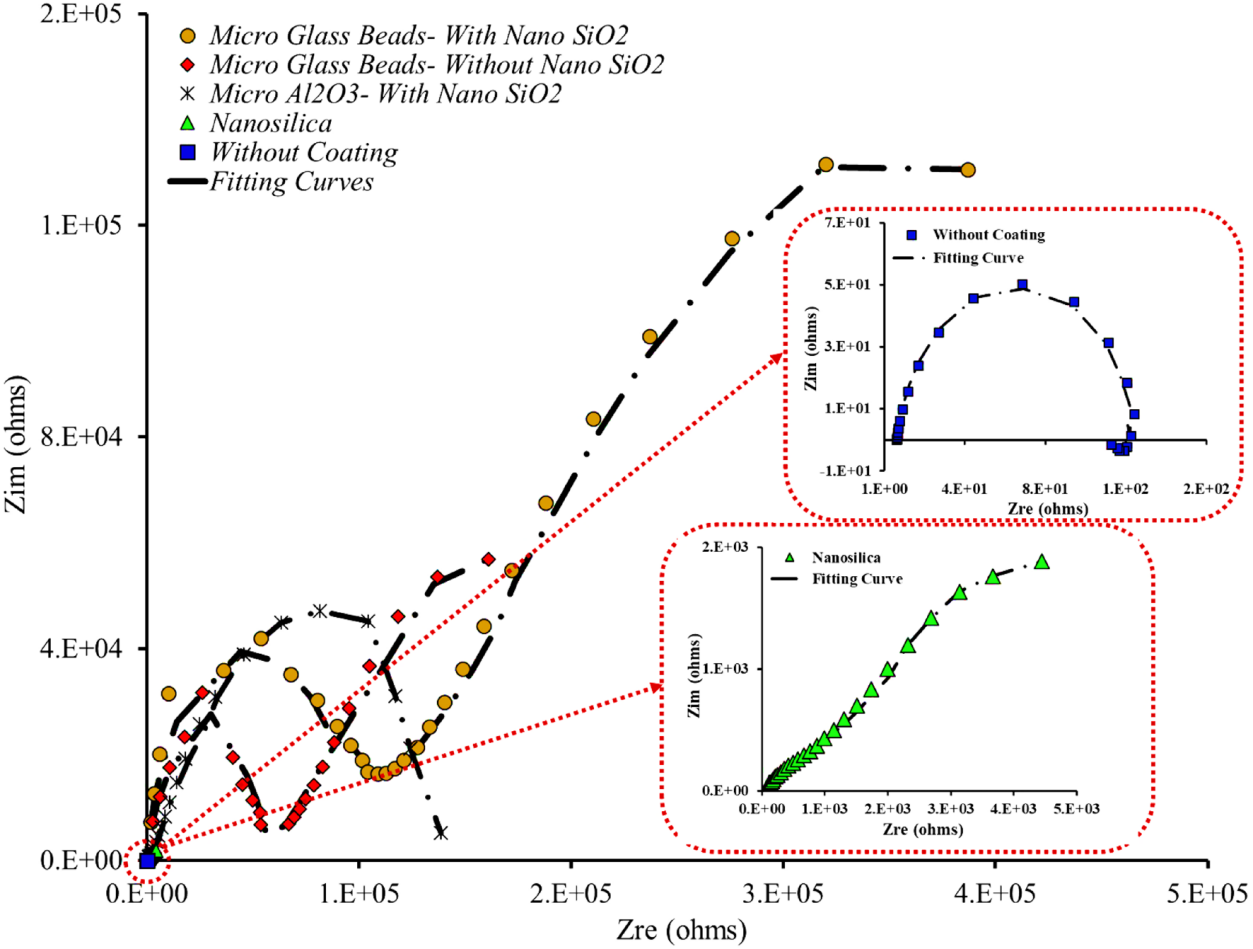
Nyquist plots for the investigated samples including: PTFE coating with glass beads microparticles in primer layer and SiO_2_ nanoparticles in overcoat layer (circle symbols), PTFE coating with glass beads microparticles in primer layer and without SiO_2_ nanoparticles in overcoat layer (diamond symbols), PTFE coating with Al_2_O_3_ microparticles in primer layer and SiO_2_ nanoparticles in overcoat layer (star symbol), nanosilica coating (triangle symbols), without coating (square symbols). Impedance spectra contain experimental data (scatter plot marked by symbols) and theoretical fitting curves (lines), which simulate the experimental results by means equivalent electrical circuits.

**Figure 7:**
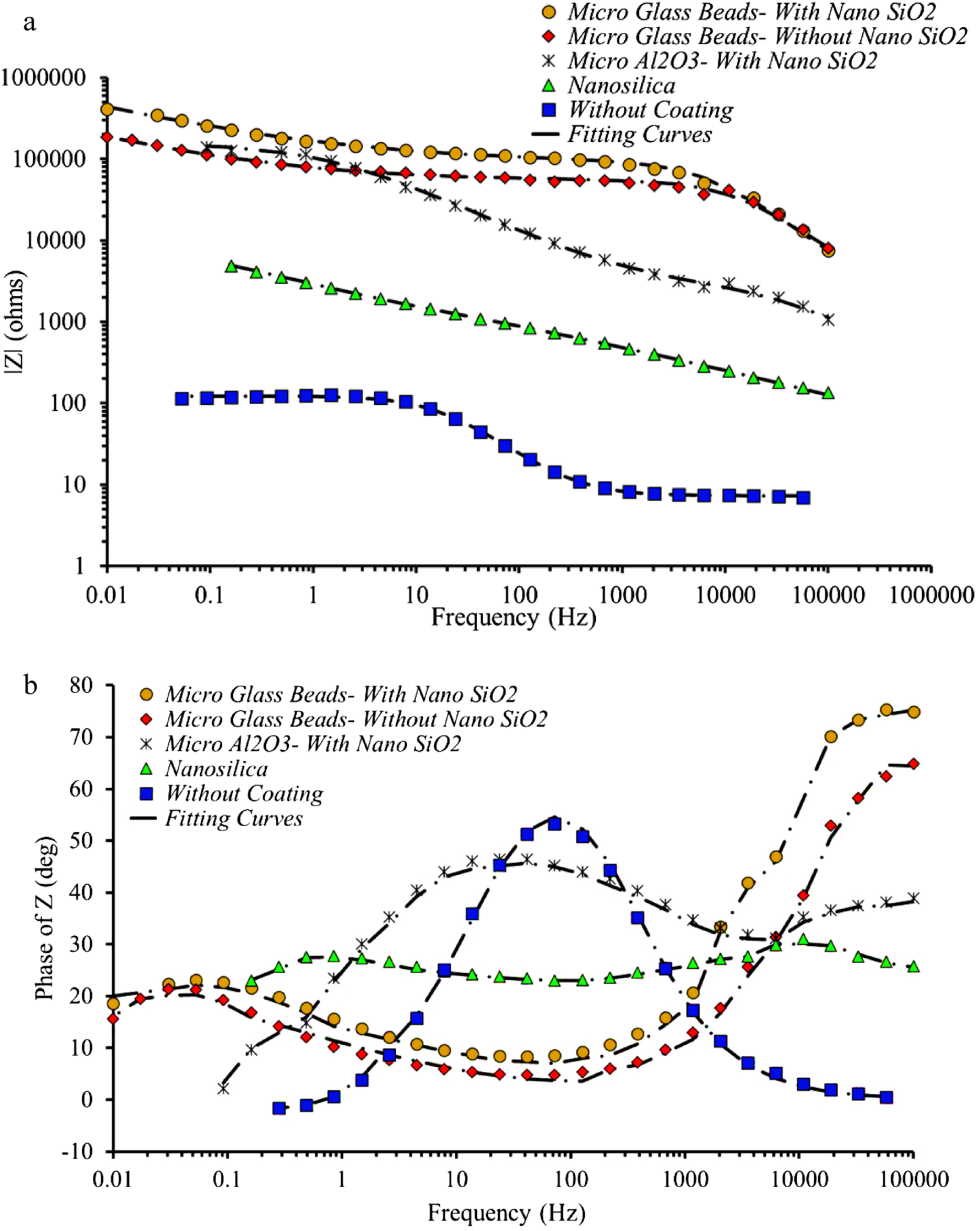
Bode (**a**) and Bode-phase (**b**) plots from EIS data of PTFE coating with glass beads microparticles in primer layer and SiO_2_ nanoparticles in overcoat layer (circle symbols), PTFE coating with glass beads microparticles in primer layer and without SiO_2_ nanoparticles in overcoat layer (diamond symbols), PTFE coating with Al_2_O_3_ microparticles in primer layer and SiO_2_ nanoparticles in overcoat layer (star symbol), nanosilica coating (triangle symbols), and uncoated sample (square symbols). Impedance spectra contain experimental data (scatter plot marked by symbols) and theoretical fitting curves (lines), which simulate the experimental results by means equivalent electrical circuits.

**Figure 8:**
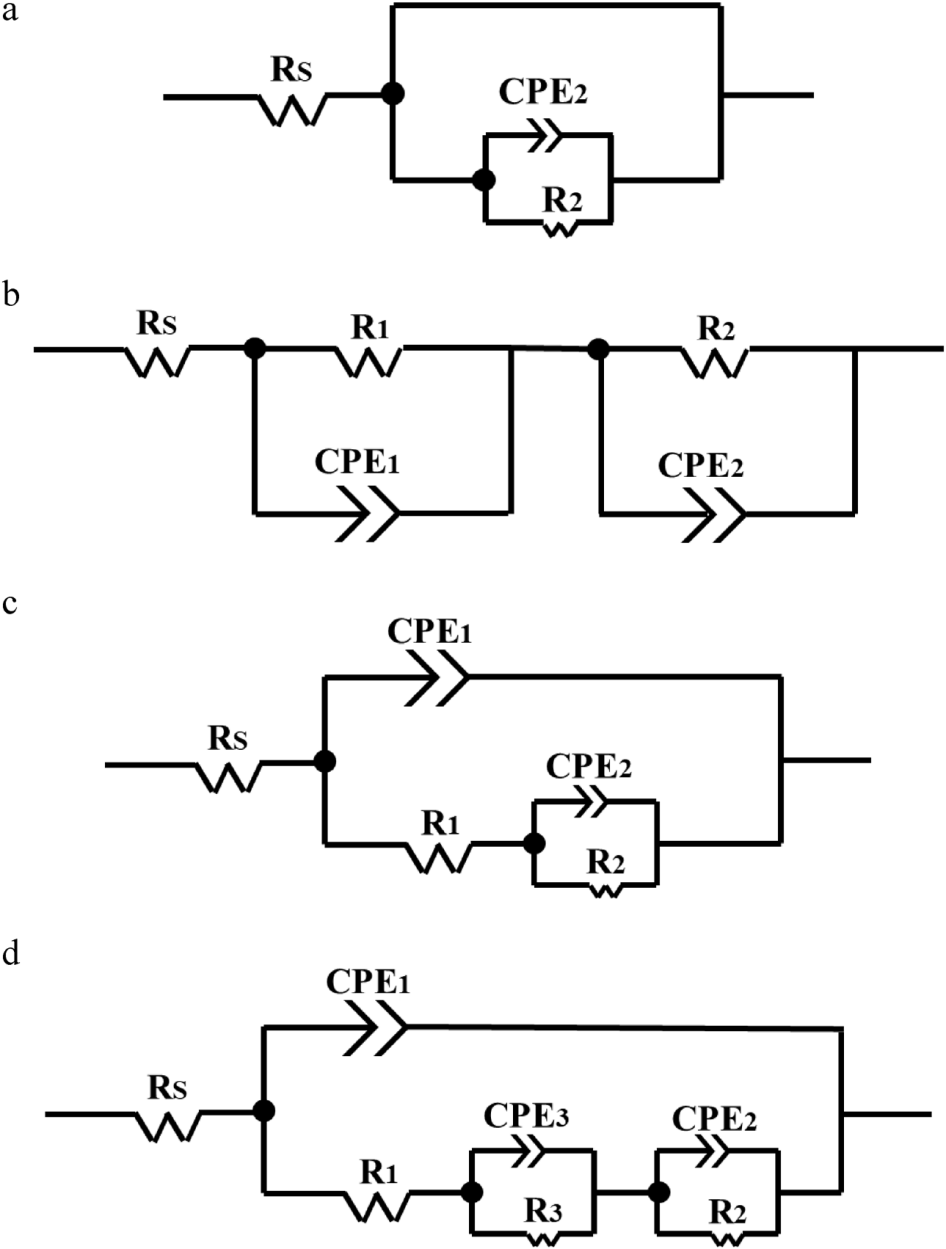
Equivalent circuit used for experimental impedance data fitting. (**a**) Uncoated sample, (**b**) nanosilica coating, (**c**, **d**) PTFE coating: (**c**) micro glass beads—without nano SiO_2_, (**d**) micro Al_2_O_3_—with nano SiO_2_ and micro glass beads—with nano SiO_2_.

In order to further investigate the corrosion behavior of the obtained coatings, the electrochemical impedance spectroscopy test was performed in 3.5% NaCl solution in open circuit potential. Nyquist plots and Bode plots for coated and uncoated samples are shown in Figs. 6 and 5, respectively. The frequency-dependent impedance modulus and phase angle graphs (Figs. 7a, b) show the characteristic changes in the morphological and electrochemical properties and the heterogeneity of the samples as a result of the formation of different layers on their surfaces^58^. The electrical equivalent circuits (EEC) used to fit the experimental data are shown in Fig. 8.

The Nyquist diagram for uncoated sample is illustrated with an inductive loop at low frequencies and a capacitive loop (semicircle) at intermediate and high frequencies (Fig. 6). The inductive performance at low frequencies is a result of adsorption of intermediate products in the pitting corrosion procedure^58^. The presence of capacitive loop is related with capacitance of the double electrical layer at the electrolyte/electrode interface and also resistance to charge transfer. The spectrum of the uncoated sample could be fitted by an EEC with one R_1_-CPE_1_ circuit (Fig. 8a). In this EEC, R_2_ is the charge transfer resistance, and CPE_2_ is a double layer capacitance. The Bode spectrum of the nanosilica coating show two-time constants (Fig. 7b). The first one with the maximum phase angle of 31.1° located near 1.08 × 10^4^ Hz and another maximum phase angle of 27.5° is located at the frequency of 4.89 × 10^–1^ Hz. The EIS spectrum measured for nanosilica coating, could be acceptably fitted with the EEC in Fig. 8b. The parameters R_1_ and CPE_1_ explain the processes performed in the coating layer and electrolyte. CPE_1_ and R_1_ are the constant phase element of the coating layer and the pore resistance due to penetration of electrolyte, respectively. The parameters R_2_ and CPE_2_ explain the processes at the substrate layer and the electrolyte interface, respectively. CPE_2_ and R_2_ are the constant phase element and the charge transfer resistance at the electrolyte/substrate layer interface, respectively. Figure 7b shows that the Bode spectrum of the PTFE coating with glass beads microparticles in the primer layer and without SiO_2_ nanoparticles in the overcoat layer also has two-time constants. The first-time constant has a maximum phase angle of 21.2° located near 5.30 × 10^–2^ Hz and the second time constant has a maximum phase angle of 62.4°, which is located near 5.74 × 10^4^ Hz. PTFE coating with glass beads microparticles in the primer layer and without SiO_2_ nanoparticles in the overcoat layer has two loops (semicircles) (Fig. 6) at high and low frequencies. In this case, both loops have capacitive properties. The spectra of this coating can be fitted by an EEC with two R-CPE circuits that is shown in Fig. 8c. In this circuit, R_1_ is coating layer resistance and R_2_ is corrosion polarization resistance, which is related to the corrosion in defective and porous areas. CPE_1_ represents the non-ideal coating capacity and CPE_2_ represents the non-ideal capacity of the double layer of electrolyte on the metal surface, the ion permeation process in the holes, and the charge transfer process at the bottom of the holes^59,60^. Considering the results obtained for this coating and the significant increase in corrosion resistance, it can be concluded that most of these pores have not reached the surface and the superhydrophobic properties have improved corrosion resistance. The quantitative parameters of the electrical equivalent circuits for PTFE coatings were calculated by fitting experimental impedance spectra using EEC with three R–CPE circuits (Fig. 8d). In this circuit, the parameters of R_1_, CPE_1_, R_2_ and CPE_2_ are coating layer resistance, non-ideal coating capacity, corrosion polarization resistance, and non-ideal capacity of the double layer of electrolyte on the metal surface, respectively. The emergence of a third time constant (R_3_-CPE_3_) could be related to better sealing up of pores on the surface of PTFE coating using SiO_2_ nanoparticles. Figures 6 and 7 show impedance spectra including experimental data and model fit curves, which simulate the experimental ones with high accuracy. There is a CPE instead of pure capacitance in the presented electrical equivalent circuits. In systems that are inhomogeneous, constant phase quantities are used instead of capacitors^48^. In other words, CPE is used to indicate processes that have some dissipative properties in addition to memory properties (such as capacitors whose charge and discharge in them is memory processes)^49^. The CPE impedance value is defined by two parameters *n* and *Q*, and its value is calculated using Eq. (2).

2 $$ Z_{CPE} = \frac{1}{{Q\left( {j\omega } \right)^{n} }} $$

where *j* is an imaginary unit, $\omega$ is angular frequency ($\omega$  =  *2πf*), *Q* and *n* are frequency independent constant and the exponential coefficient, respectively. The measured electrical equivalent circuit parameters obtained with fitting appropriate circuits to the experimental EIS data are reported in Table 5.

**Table 5:** Equivalent circuit parameters obtained by modeling the data of the coatings impedance spectroscopy.

Sample	R_1_ (Ω cm^2^)	CPE_1_		R_2_ (Ω cm^2^)	CPE_2_		R_3_ (Ω cm^2^)	CPE_3_	
		Q_1_ (/Ω cm^2 ^S^n^)	n_1_		Q_2_ (/Ω cm^2 ^S^n^)	n_2_		Q_3_ (/Ω cm^2 ^S^n^)	n_3_
Without coating	–	–	–	118.5	1.5 × 10^–4^	0.88	–	–	–
Nanosilica	1483	1.06 × 10^–4^	0.32	1.17 × 10^+4^	1.96 × 10^–4^	0.48	–	–	–
Micro Al_2_O_3_—with nano SiO_2_	2137	1.01 × 10^–8^	0.86	5.71 × 10^+4^	2.29 × 10^–6^	0.56	8.28 × 10^+4^	1.64 × 10^–6^	0.88
Micro glass beads—without nano SiO_2_	5.59 × 10^+4^	1.06 × 10–9	0.86	2.29 × 10^+6^	1.91 × 10^–5^	0.38	–	–	–
Micro glass beads—with nano SiO_2_	6.05 × 10^+4^	3.01 × 10^–10^	0.97	4.52 × 10^+4^	3.36 × 10^–8^	0.69	8.54 × 10^+5^	6.51 × 10^–6^	0.92

According to Fig. 7a, it can be concluded that the superhydrophobic coatings fabricated in this study (especially PTFE coatings) have led to a significant increase in the impedance modulus *|Z|*f → 0 Hz, compared to the uncoated sample. The high value of the impedance modulus at low frequency, *|Z|*f → 0 Hz, indicates the high protective characteristics for superhydrophobic coatings. It is observed that PTFE coating with glass beads microparticles in the primer layer and SiO_2_ nanoparticles in the overcoat layer has better performance than other samples. The electrode/electrolyte interface for this sample has a capacitive character according to the behavior of the impedance spectra. This consequence shows that the coating is homogeneous and there is no cracks and defects in its structure. Obviously, all these features are due to sealing up of defects and pores in this coating. After this coating, PTFE coatings with glass beads microparticles in the primer layer and without SiO_2_ nanoparticles in the overcoat layer, PTFE coatings with Al_2_O_3_ microparticles in the primer layer and with SiO_2_ nanoparticles in the overcoat layer, and finally nanosilica coating are placed according to their performance, respectively. Examination of EECs parameters (Table 5) for coated samples shows an increase in R_1_ and a decrease in Q_1_ (these parameters determine the porous layers of the coating). As can be seen, PTFE coatings have more R_1_ and less Q_1_. This could be due to the increased thickness of the coating as a result of the application of micro-nanoparticles as well as PTFE coating layers compared to nanosilica coating. A comparison of the results obtained in Table 5 shows that among PTFE coatings, the sample with glass beads microparticles in the primer layer and SiO_2_ nanoparticles in the overcoat layer has the highest R_1_ and the lowest Q_1_. The increase in exponential coefficient (*n*1) indicates an increase in the homogeneity of PTFE coating with glass beads microparticles in the primer layer and SiO_2_ nanoparticles in the overcoat layer, compared to the other two types of PTFE coatings as well as nanosilica coating. The high value of the electrical resistance R_3_ and the low value Q_3_ for two PTFE coatings, including PTFE coatings with Al_2_O_3_ microparticles in the primer layer and SiO_2_ nanoparticles in the overcoat layer, and PTFE coatings with glass beads microparticles in the primer layer and SiO_2_ nanoparticles in the overcoat layer (Table 5) prove that these two coatings are homogeneous. For these two coatings, the exponential coefficient (*n*3) is equal to 0.88 and 0.92, respectively. This shows that these two coatings are very homogeneous and the pores in the coating are well closed by applying SiO_2_ nanoparticles in the overcoat layer. Based on the results obtained from Table 5, it can be concluded that, the PTFE coating with the glass beads microparticles have much higher resistance than the other two samples. Also, among two samples with glass beads microparticles, the sample containing SiO_2_ nanoparticles has more resistance than the sample without SiO_2_ nanoparticles, and this confirms what mentioned in the preceding sections.

The results of this study suggest that reducing the surface area in contact with corrosive solutions can be a very effective way to increase corrosion resistance. Bico et al.^61^ attributed the imprisonment of air bubbles to holes and heights of surface as a factor in creating a quasi-stable state, according to Eq. (3).

3 $$ \cos \theta \le \frac{{f_{1} - 1}}{{\gamma - f_{1} }} $$

where θ is CA, γ is surface roughness rate, and *f1* the fraction of the solid/liquid interface in contact with the droplet. Based on this equation, if θ is greater than 90°, air bubbles can be trapped in the solid/liquid interface. It has also been reported that when θ is greater than 90°, the possibility of absorption of corrosive species such as Cl^−^ ions on solid surfaces is reduced and corrosion resistance is greatly increased. The coatings synthesized in this study have superhydrophobic properties and a combination of superhydrophobic properties with low electrical conductivity materials significantly increased corrosion resistance. The results of the EIS test confirm the accuracy of the polarization test results. It should be noted that the numbers obtained for the resistance in both tests are not the same, but their changes are similar. The mismatch of numbers can be attributed to the occurrence of uneven corrosion (to calculate the R_P_, the corrosion must be uniform), as well as the error of using the equivalent circuit.

## Conclusions

In this study, the corrosion behavior of different samples including uncoated sample, nanosilica coating, PTFE coating with Al_2_O_3_ microparticles in the primer layer and SiO_2_ nanoparticles in the overcoat layer, PTFE coatings with glass beads microparticles in the primer layer and an overcoat with and without SiO_2_ nanoparticles, were analyzed by TOEFL polarization and EIS tests in 3.5% NaCl solution. The results of this study are as follows:

1. The corrosion resistance of all coated samples is much higher than that of uncoated samples and among them the PTFE coating with glass beads microparticles has the highest corrosion resistance.
2. Electrical resistance and penetration rate are two important issues in the corrosion behavior of the specimens. Increasing the thickness of the coatings, decreasing the amount of electrolyte penetration into the coating and also the insulation of the coatings, increase the corrosion resistance. In this regard, PTFE coating with glass beads microparticles has higher corrosion resistance than silica film coating and PTFE coating with Al_2_O_3_ microparticles. This could be attributed to lower thickness of the silica film and conductivity of Al_2_O_3_ powder. Also, PTFE coating with glass beads microparticles in primer layer and SiO_2_ nanoparticles in overcoat layer has higher corrosion resistance than PTFE coating with glass beads microparticles in primer layer and without SiO_2_ nanoparticles in the overcoat layer.
3. Superhydrophobic property along with low conductivity feature is an important factor in increasing corrosion resistance. Also, the presence of SiO_2_ nanoparticles in PTFE superhydrophobic coating improves corrosion protection properties by sealing up the defects and pores in the coating. In this study, PTFE coating with glass beads microparticles in primer layer and SiO_2_ nanoparticles in overcoat layer (the best coating obtained in this study) compared to the uncoated sample, reduced the corrosion rate by almost 80 times.

## Data Availability

All data generated or analysed during this study are included in this article.
